# Impact of image plane selection on measures of left ventricular non-compaction

**DOI:** 10.1186/1532-429X-16-S1-P274

**Published:** 2014-01-16

**Authors:** Filip Zemrak, Nadine Kawel-Boehm, Saidi A Mohiddin, Mark Westwood, Neha Sekhri, Roshan Weerackody, Ceri Davies, Francesca Pugliese, Steffen E Petersen

**Affiliations:** 1 Centre of Advanced Cardiovascular Imaging, The London Chest Hospital, London, UK; 2 University Hospital Basel, Basel, Swaziland

## Background

Cardiac magnetic resonance (CMR) is frequently used to assess the extent of trabeculations in cases of left ventricular non-compaction. Most routine CMR studies do not acquire full 3D isotropic cine datasets and consequently suboptimal acquisition of prescribed image views could lead to clinically important inaccuracies in calculating non-compacted to compacted myocardial ratios (NC/C). This study compares measurement of the NC/C ratio between optimal and off-axis views derived from 3D cardiac computer tomography (CT) datasets and compares these with those derived from optimal CMR datasets.

## Methods

We studied 10 participants who underwent CMR and contrast CT imaging (retrospective gating) as part of the Evaluation of Integrated Cardiac Imaging for the Detection, Characterization and Monitoring of Ischemic Heart Disease study protocol (EVINCI study). NC/C ratios were determined for CMR and for CT images in mid and apical anterior and inferior segments based on 2 chamber views and short axis views in diastole. NC/C ratios in the same segments were compared in "off-axis" 2-chamber view and short axis views in optimal position and tilted by 30 and 45 degrees for CT.

## Results

Comparing measurements from optimal 2-chamber views for CMR and CT, there was no difference for NC/C ratio (CMR 1.1 ± 0.5 vs. CT 0.8 ± 0.3, p = 0.2). The NC/C ratio was higher in MRI short axis view compared with CT short axis (1.0 ± 0.4 vs. 0.6 ± 0.4, p = 0.001). The NC/C ratio was overestimated from off-axis 2 chamber CT views compared to the optimal 2 chamber view: NC/C 1.0 ± 0.4 vs. 0.8 ± 0.3, p = 0.001. There were no differences between NC/C ratio in optimally oriented short axis and angulated planes: 0.6 ± 0.4 vs. 0.6 ± 0.3 (30 degrees) vs. 0.6 ± 0.3 (45 degrees), p = 0.9.

## Conclusions

Diagnostic cut-off values for NC/C are likely to be lower for CT compared to CMR, particularly if measured in the short axis slices. NC/C ratios are overestimated in the 2 chamber view when the plane is off axis. NC/C ratios determined in short axis views are less sensitive to angulation of the image plane and may thus be preferable if long axis images are not ideally positioned, however with separate cut-off values for this plane.

## Funding

NIHR Cardiovascular Biomedical Research Unit.

